# Genome-wide analysis and identification of microRNAs in *Medicago truncatula* under aluminum stress

**DOI:** 10.3389/fpls.2023.1137764

**Published:** 2023-01-27

**Authors:** Zhongjie Lu, Zhengyu Yang, Zheng Tian, Qihui Gui, Rui Dong, Chao Chen

**Affiliations:** ^1^ Department of Grassland Science, College of Animal Science, Guizhou University, Guiyang, China; ^2^ Department of Vehicle Engineering, Guizhou Technological College of Machinery and Electricity, Duyun, China

**Keywords:** plant miRNA, *Medicago truncatula*, aluminum stress, transcript analysis, plant growth

## Abstract

Numerous studies have shown that plant microRNAs (miRNAs) play key roles in plant growth and development, as well as in response to biotic and abiotic stresses; however, the role of miRNA in legumes under aluminum (Al) stress have rarely been reported. Therefore, here, we aimed to investigate the role of miRNAs in and their mechanism of Al tolerance in legumes. To this end, we sequenced a 12-strand-specific library of *Medicago truncatula* under Al stress. A total of 195.80 M clean reads were obtained, and 876 miRNAs were identified, of which, 673 were known miRNAs and 203 were unknown. A total of 55 miRNAs and their corresponding 2,502 target genes were differentially expressed at various time points during Al stress. Further analysis revealed that mtr-miR156g-3p was the only miRNA that was significantly upregulated at all time points under Al stress and could directly regulate the expression of genes associated with root cell growth. Three miRNAs, novel_miR_135, novel_miR_182, and novel_miR_36, simultaneously regulated the expression of four Al-tolerant transcription factors, GRAS, MYB, WRKY, and bHLH, at an early stage of Al stress, indicating a response to Al stress. In addition, legume-specific miR2119 and miR5213 were involved in the tolerance mechanism to Al stress by regulating F-box proteins that have protective effects against stress. Our results contribute to an improved understanding of the role of miRNAs in Al stress in legumes and provide a basis for studying the molecular mechanisms of Al stress regulation.

## 1 Introduction

Aluminum (Al) toxicity is one of the most important factors limiting crop growth and production in acidic soils. Approximately 40% of global arable land is acidic, which is one of the main factors contributing to food shortages in regions such as those in Africa (Daspute et al., 2017). Al^3+^ is present in naturally acidic soils and is also the most harmful form of Al to plants, with micromolar concentrations of Al^3+^ inhibiting root elongation in a short period of time (Gui et al., 2022). Chandran et al. (2008) stained the root tips of *Medicago truncatula* seedlings grown at 10-μm Al concentration with hematoxylin and showed that the root tips treated with Al for the longest time stained the darkest, indicating that they were the most severely damaged. Li et al. (2020) found that Al stress significantly enhanced the expression of *MsPG1* in the plasma membrane of *Medicago sativa* root apical epidermal cells, reduced the accumulation of Al in the cell wall, and improved the Al tolerance of *M. sativa*. However, some plants grown for a long time in acidic soils can chelate Al^3+^ in the vicinity of the root zone with organic acids such as malic, citric, and oxalic acids secreted by the root system, forming Al-organic acid complexes, and thus reducing the transport of Al to the interior of the root cell and enhancing Al tolerance (Chauhan et al., 2021).

MicroRNAs are a subset of the major non-coding RNAs in higher plants. They are typically 19–24 nucleotides long, and they are thought to post-transcriptionally regulate the cleavage of target mRNAs or repress their translation (Bartel, 2004; Kumar et al., 2017; Xu et al., 2019). miRNAs play key roles in gene expression, stress responses, growth and development, and other regulatory mechanisms in plants and animals (Zhang et al., 2015; Pegler et al., 2019). Liu et al. (2014) studied 22 conserved miRNA families in *Zea mays* and found that 72 genes targeted by 62 differentially expressed miRNAs may regulate maize ear development. Wang et al. (2011) identified two miRNAs, cbr-mir-241 and ath-miR854a, using microarray technology, that can directly regulate *Glycine max* resistance to blast rot through their targets (enzymes). Hoang et al. (2020) found that miRNAs play an important role in various processes of symbiotic nitrogen fixation with rhizobia in four legumes: *Lotus japonicus*, *M. truncatula*, *G. max*, and *Phaseolus vulgaris*.

*Medicago truncatula* is an annual legume that has been used as a model plant for legumes as it is a close relative of *M. sativa* and has the advantage of having a small genome, high similarity, and being a diploid plant (Chandran et al., 2008). In recent years, although researchers have conducted numerous studies on Al stress in plants, miRNA studies related to Al stress in legumes have rarely been reported. Therefore, we aimed to investigate the role of miRNAs in and their mechanism of Al tolerance in *M. truncatula*. High-throughput sequencing of root tip tissues under Al stress was performed to identify miRNAs associated with the Al stress response. The findings of this study provide new insights into the potential functions of miRNAs in the Al response mechanism.

## 2 Materials and methods

### 2.1 Plant materials and processing

*Medicago truncatula* A17 seeds were surface-disinfected with 1% NaClO solution for 10 min and rinsed five times in distilled water to remove residual disinfectant solution, then incubated in an artificial climate chamber at 25°C for 3 days protected from light, and then transferred to Hoagland’s medium (pH 5.8) for 7 days (25°C, light/dark cycle of 16/8 h). The composition of Hoagland’s culture solution is consistent with the experiments of Stephan and Procházka (1989).The nutrient solution was changed every 2 days during incubation. Seedlings with similar growth levels were divided into four groups, three of which were incubated in 10 μM AlCl_3_ and 0.5 mM CaCl_2_ (pH 4.5) solutions for 4, 24, and 48 h, respectively, and were recorded as T4, T24, and T48, respectively. The control group (T0) was incubated in a 0.5 mM CaCl_2_ (pH 4.5) solution for 48 h. To treat the four groups of seedlings, two groups of seedlings, T0 and T48, were incubated simultaneously. After 24 h and 44 h of incubation of the above two groups of seedlings, respectively, the seedlings of T24 and T4 groups were incubated, and the four groups of seedlings were harvested simultaneously at 48 h to reduce the circadian effect. Three replicates were set up for each group, with 60 seedlings per replicate. After treatment, root tips of ~ 1.5 cm from each treated plant were collected, immediately frozen in liquid nitrogen, and stored at –80 °C.

### 2.2 Physiological indicators and fluorescein diacetate staining test

The activities of three enzymes, superoxide dismutase (SOD, SOD-BC0170), catalase (CAT, CAT-BC0200), and peroxidase (POD, POD-BC0090), were measured in four sets of samples using kits provided by Beijing Solarbio Science and Technology Co., Ltd. (Beijing, China). The specific operational steps of the test are presented in the instruction manual.

An image scanner (Perfection V800 photo, Epson, Suwa, Japan) with a WinRhizo root analysis system (WinRhizo Tron Pro 2009, Regent Instruments Inc., Quebec, Canada) was used to measure the root length and root surface area of four sets of samples (Pang et al., 2018). When treating root length and root surface area, we included a control group of plant material grown under Al-free conditions in a 0.5 mM CaCl_2_ solution (pH 4.5) for the same duration as that of the corresponding Al treatment. The root vigor assay was performed using the naphthylamine method (BC5295, Beijing Solarbio Science and Technology Co., Ltd., Beijing, China). Sample root tips were treated in 2 g/mL FDA solution protected from light for 10 min according to the method of Ishikawa and Wagatsum (1998). The treated root tips were repeatedly rinsed at least five times with deionized water, followed by observation and photography using a fluorescence microscope (Axiolab5, ZEISS, Germany). Each sample was repeated three times. The aforementioned experimental operations were performed under dark conditions.

### 2.3 RNA extraction, library preparation of sRNA, and sequencing

RNA samples were extracted using TRIzol (Invitrogen, Carlsbad, CA, USA) and tested for concentration, purity, and integrity (Liu et al., 2016). The resulting 3**′** sRNA and 5**′** sRNA were ligated for splicing. The first strand was synthesized by reverse transcription. Finally, polymerase chain reaction (PCR) amplification and size selection were performed. The target fragments were screened by PAGE, and the cut gels were recovered as fragments to obtain sRNA libraries. Finally, the PCR products were purified (AMPure XP system) and the library quality was assessed.

The resulting libraries were sequenced on the Illumina NovaSeq 6,000 platform from Biomarker Co., Ltd. (BMKcloud, Beijing, China). Clean data were obtained by removing reads containing adapters, poly-N, and low-quality reads from the raw data (Zhao et al., 2020). Using Bowtie tools, clean reads were aligned against Silva and other databases to screen for other sRNAs, such as ribosomal RNA (rRNA), which were then eliminated. Finally, the unannotated reads were sequenced against the reference genome Medicago_truncatula.Mt4.0v2 using Bowtie2 (v1.0.0) software to obtain information on the position of the reads in the reference genome (Pertea et al., 2015).

### 2.4 Identification of miRNAs and prediction of new miRNAs

The reads that were aligned to the reference genome were then compared to the mature sequences of known miRNAs in the miRBase (v22) database and their upstream 2nt and downstream 5nt ranges, and the identified reads were considered known miRNAs. As miRNA precursors have a signature hairpin structure, the formation of the mature body is achieved by shearing of the Dicer/DCL enzyme. Based on these features, the miRDeep2 software package (Friedlander et al., 2012) was used, and the final prediction of new miRNAs was achieved by scoring using a Bayesian model (Zhang, 2015).

### 2.5 Analysis of differentially expressed miRNAs

Differential expression analysis of the two conditions/groups was performed using the DESeq2 R package (1.10.1) (Love et al., 2014). The resulting *P-values* were adjusted using Benjamini and Hochberg’s approach to control for the false discovery rate. miRNAs with |log2(FC)| ≥ 0.58; *P-value* ≤ 0.05, found by DESeq2, were assigned as differentially expressed. Fold Change (FC) indicates the ratio of expression between two samples (groups).

### 2.6 Prediction, annotation, and functional analysis of DE miRNAs target genes

Target gene prediction was performed using TargetFinder (v1.6) software based on the gene sequence information of known miRNAs and newly predicted miRNAs in the corresponding species (Allen et al., 2005). The predicted target gene sequences were compared with the NCBI non-redundant protein sequence (Nr), SwissProt Protein databases, Gene Ontology database (GO), Clusters of Orthologous Groups of proteins (COG), Kyoto Encyclopedia of Genes and Genomes (KEGG), Clusters of Protein homology (KOG), Evolutionary genealogy of genes (eggNOG), and protein family (Pfam) (*E-value* < 10^-5^) (Yu et al., 2022) databases using BLAST (v2.2.26) software to obtain the annotation information of the target genes. GO enrichment and KEGG enrichment of miRNA target genes were analyzed using Cluster Profiler (v3.10.1) software and KOBAS software (Mao et al., 2005), respectively.

### 2.7 Quantitative real-time polymerase chain reaction analysis

The extracted total RNA, combined with reverse random primers, was used for reverse transcription of miRNA. The qRT-PCR was performed using the MyiQ Single Color Real-time PCR system (Bio-Rad, Hercules, CA, USA) (95°C for 3 min and 45 cycles of 95°C for 5 s and 60°C for 30 s). Primers were designed using Primer Premier (v6.0) software and synthesized by Sangon Biotech Co., Ltd. (Shanghai, China) (**Supplementary Table S1**). The 2^-ΔΔCt^ method was used to the calculate relative gene expression levels.

## 3 Results

### 3.1 Physiological characteristics of roots under Al stress

**Figure 1:**
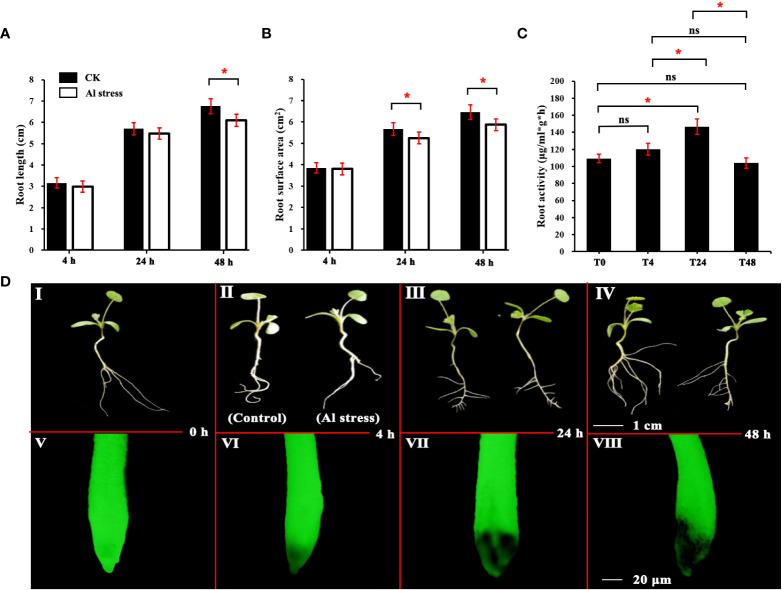
Analysis of root physiological characteristics of *M. truncatula* under aluminum stress. **(A)** Analysis of total root length of *M. truncatula*. **(B)** Analysis of root surface area of *M. Truncatula*. **(C)** Analysis of root activity of *M. Truncatula*. **(D)** I–IV is the growth comparison of plants under Al stress and the blank control at the same time point. Plant material grown under normal conditions and treated for the same amount of time as the corresponding aluminum treatment group were used as a control, scale = 1 cm; V–VIII is the comparison of root fluorescence of plants under Al stress and the blank control after FDA staining, scale = 20 μm. * indicates that there is a significant difference at the level of 0.05 according to Duncan’s multiple range test, while ns indicates that the difference is not significant.

The root length, root surface area, root activity, and plant growth of *M. truncatula* plants after Al treatment are shown in **Figures 1A–C**. With increasing duration of Al stress, the root length of plants in the Al-treated group differed significantly from that of the control at 48 h (**Figure 1A**), and their root length decreased by 5.0% compared with the control. The plant heel surface area of the Al-treated group was significantly different from that of the control group at both 24 and 48 h (**Figure 1B**), with reductions of 3.9% and 4.5%, respectively. Root activity showed an increasing trend and then decreasing, and was significantly lower at 48 h than at 24 h (**Figure 1C**). As shown in **Figure 1D** (I–IV), the root morphology of the Al-treated and control plants also showed significant differences as the stress time increased.

The FDA fluorescent staining method only stains live cells. Therefore, the FDA staining of the root system at each treatment time point revealed that damage to the plant root tip tissues had already begun after 4 h of Al treatment, and the damage to the root-tip tissues gradually increased with the Al treatment time (**Figure 1D** V–VIII).

**Figure 2:**
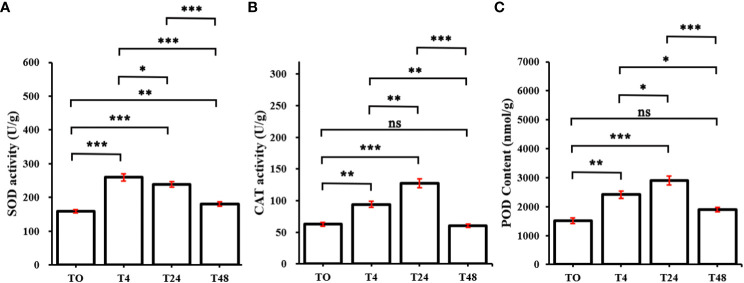
Changes of oxidoreductase in *M. truncatula* under continuous aluminum stress. **(A)** superoxide dismutase (SOD) activity. **(B)** Catalase (CAT) activity. **(C)** peroxidase activity (POD). The plotting data is the average and standard deviation of three repetitions. T0~T48 represent *M. truncatula* plant samples treated with aluminum for 0h, 4h, 24h, and 48h, respectively. According to Duncan’s multiple range test, the asterisk represents the significant difference between aluminum treated samples and the control (*, *P* < 0.05; **, *P* < 0.01; ***, *P* < 0.001), ns indicates that the difference is not significant.

To investigate whether the redox system was activated in *M. truncatula* subjected to Al stress, the enzymatic activities of SOD, CAT, and POD were examined in this study. The SOD activity reached its maximum at 4 h, after which, it started to decline, and decreased significantly at 48 h (**Figure 2A**). CAT and POD showed the same trend, with both significantly increasing at 4 h versus 24 h and significantly decreasing at 48 h (**Figures 2B, C**). These results indicated that the redox system in *M. truncatula* was rapidly activated under Al stress.

### 3.2 MicroRNA sequence analysis and mapping

In this study, 12 *M. truncatula* samples were sequenced for small RNA and a total of 296.01 M raw reads were obtained. After removing contaminants and reads of length < 18 and > 30 nucleotides, 195.80 M clean reads were obtained. There were not less than 11.96 M clean reads for each sample, the average GC content and base number were 50.99% and 1258.05 Mb, respectively, and the average Q30 was 86.82% (**Supplementary Table S2**). The average mapped reads, uniquely mapped reads, and multiple-mapped reads constituted 61.46%, 40.42%, and 21.05% of all libraries, respectively, and the comparison efficiency of the reads in each sample against the reference genome ranged from 51.76–68.47% (**Supplementary Table S3**). The length distribution of sRNAs was similar among the 12 libraries, with the highest abundance of 21-nucleotide sRNAs, followed by 24-nucleotide sRNAs (**Supplementary Table S4**), which is consistent with the previous findings of Bao et al. (2019).

### 3.3 Identification of known and novel miRNAs, predictive analysis of target genes, and functional annotation

A total of 876 miRNAs were identified in this study, of which, 673 were known and 203 were unknown. There were 556 known miRNAs with family affiliation and 95 unknown miRNAs with family affiliation (**Supplementary Table S5**). Subsequently, miRNA target genes were predicted using TargetFinder software; 664 of the known miRNAs predicted 10,845 target genes, and 194 of the unknown miRNAs predicted 3,610 target genes (**Supplementary Table S6**). According to our functional annotation results, a total of 13,057 out of 13,148 target genes were annotated, with the number ranging from 3,852 (29.50%, KEGG) to 13,055 (99.98%, Nr), among which, the most genes were annotated in Nr and eggNOG with 13,055 and 10,935, respectively (**Supplementary Table S7**).

### 3.4 MicroRNA differential expression analysis, predictive analysis, and functional annotation of target genes

In total, 55 differentially expressed miRNAs (**Supplementary Table S8**) were identified in this study. Comparison of the T0 *vs*. T4 treatment groups showed the highest number of differentially expressed miRNAs, 48, of which, 31 were upregulated and 17 were downregulated. Five miRNAs were differentially expressed in the comparison between the T0 *vs*. T24 treatment groups, with all of them exhibiting upregulation. Ten miRNAs were differentially expressed in the comparison between the T0 *vs*. T48 treatment groups, of which, five were upregulated and five were downregulated. A total of 2,502 differential target genes were predicted for the 55 differentially expressed miRNAs; the maximum number of target genes of differentially expressed miRNAs was 2,088 in the T0 *vs*. T4 treatment group and the minimum was 101 in the T0 *vs*. T24 treatment group. Subsequently, the target genes of the differentially expressed miRNAs were annotated using eight databases, including Nr, KOG, COG, Pfam, Swiss-Prot, eggNOG, GO, and KEGG, and all 2,502 target genes were annotated. The target genes of all three treatment groups were the most abundant annotated genes in the NR database, with 2088, 101, and 307, respectively (**Supplementary Table S9**).

### 3.5 Gene ontology function and KEGG pathway enrichment analysis

**Figure 3:**
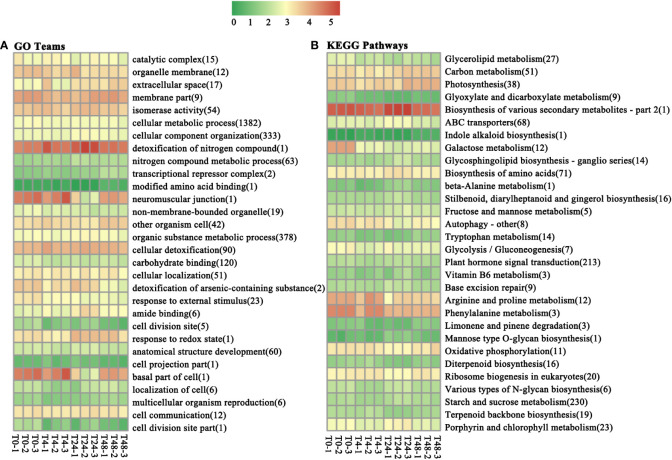
Under aluminum stress, 30 GO terms and KEGG pathways were enriched for the most DEGs. **(A)** GO teams. **(B)** KEGG pathways. The numbers in the abscissa represent the number of DEGs enriched for the GO term or KEGG pathway. T0 to T48 represent *M. truncatula* plant samples treated with aluminum for 0h, 4h, 24h, and 48h, respectively. The comparison is described as A *vs* B, which means the DEG was found in the A treatment relative to the B treatment.

To determine whether miRNAs are functionally involved in the Al stress response and defense processes, we performed GO functional analysis of differential target genes of miRNAs. A total of 2,714 DEGs were enriched in the 145 GO treatments (**Supplementary Table S10**). Based on the functions of the enriched genes in each GO tree, a cluster analysis of 145 GO terms could be clustered into 30 major classes (**Figure 3A**). The three GO terms “Cellular metabolic process,” “Organic substance metabolic process,” and “Cellular component organization” had the highest number of DEGs, at 1,382, 378, and 333, respectively (**Figure 3A**).

KEGG pathway enrichment was performed to explore the function of genes differentially expressed under Al stress as well as metabolic pathways involved in the response. A total of 912 DEGs were enriched in 111 pathways (**Supplementary Table S9**). The 111 enriched pathways were clustered into 30 broad categories based on the function of the enriched genes in each pathway (**Figure 3B**). The highest number of DEGs was found in three enrichment pathways: “starch and sucrose metabolism,” “plant hormone signal transduction,” and “biosynthesis of amino acids,” at 230, 213, and 71, respectively.

### 3.6 qRT-PCR verification of DE miRNA and differential genes

**Figure 4:**
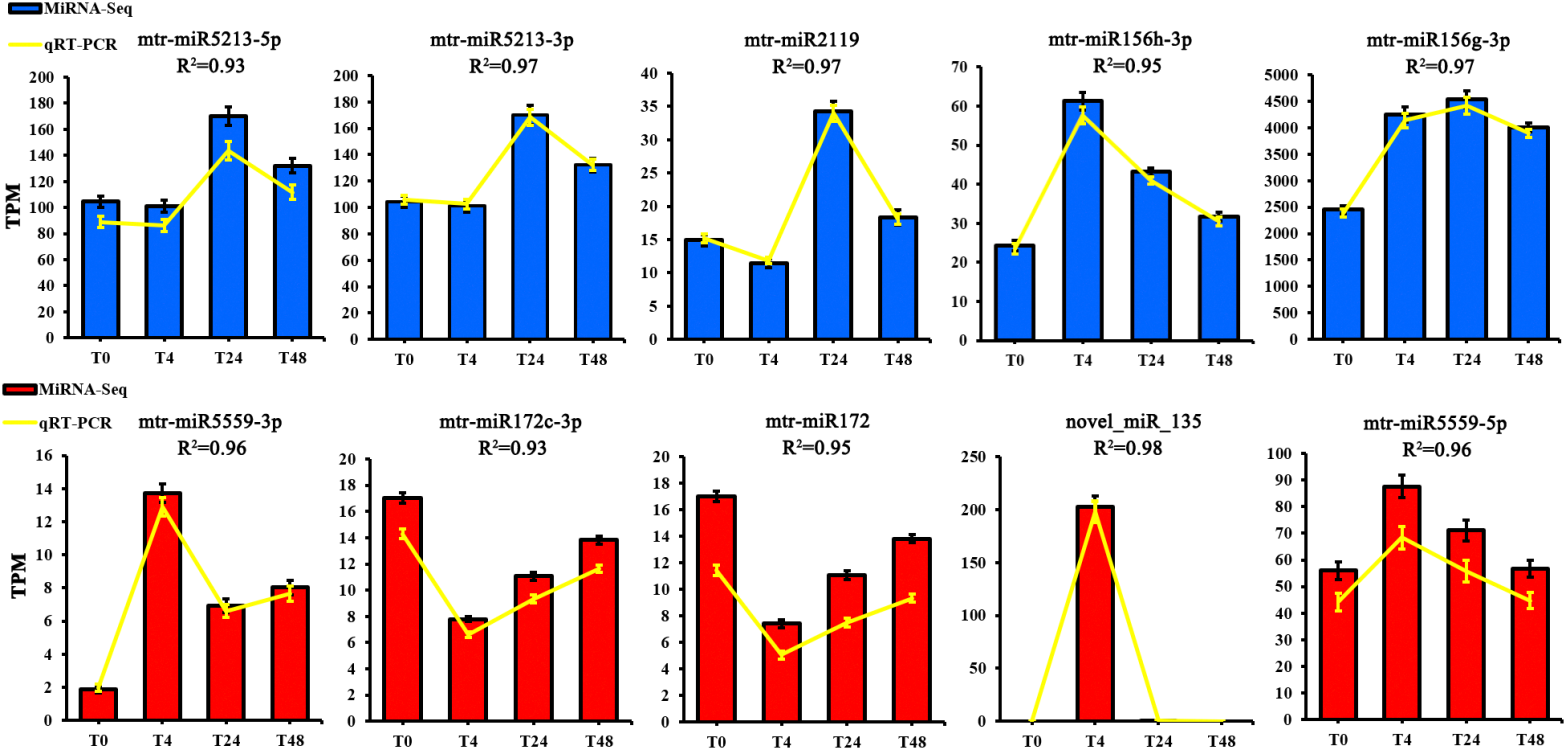
The expression patterns of ten selected miRNAs identified by RNA-Seq were verified by qRT-PCR. The Y-axis to the left of each histogram represents the expression level of RNA-seq (TPM). T0 to T48 represents *M. truncatula* plant samples treated with aluminum for 0h, 4h, 24h, and 48h, respectively.

Ten DE miRNAs were randomly selected for qRT-PCR validation to verify the reliability of the transcriptome data. The results showed that these miRNAs had similar expression levels and trends as observed in the miRNA-seq results (**Figure 4**) and the qRT-PCR results also demonstrated the reliability of the transcriptome data.

## 4 Discussion

Al stress contributes majorly to altered metabolic activity, root damage, cell wall damage, and cytoplasmic lysis, resulting in reduced photosynthetic efficiency, impaired water and nutrient uptake, and increased respiratory consumption and toxin accumulation; these effects limit crop growth (Xu et al., 2021), ultimately leading to plant death and yield loss (Osman et al., 2021; Abdel Latef et al., 2021). MicroRNAs widely regulate various physiological processes, such as development, signal transduction, and stress response in plants (Chen, 2012; Sunkar et al., 2012), and their mechanisms in plant stress tolerance have been demonstrated in *G.max* (Ning et al., 2019), *Z. mays* (Fu et al., 2017; Shan et al., 2020), and *Arabidopsis thaliana* (Shukla et al., 2018).

In this study, a total of 12 sRNA samples of *M. truncatula* under Al stress for 4 h, 24 h, 48 h and a control group were subjected to whole transcriptome sequencing, and a total of 876 miRNAs were obtained, among which, 203 were unknown miRNAs. Cao et al. (2018) similarly identified 876 miRNAs under salt/alkali stress in *M. truncatula*, supporting the reliability of the data in this study.

### 4.1 Effect of mtr-miR156g-3p on the root system of *M. truncatula* under Al stress

**Figure 5:**
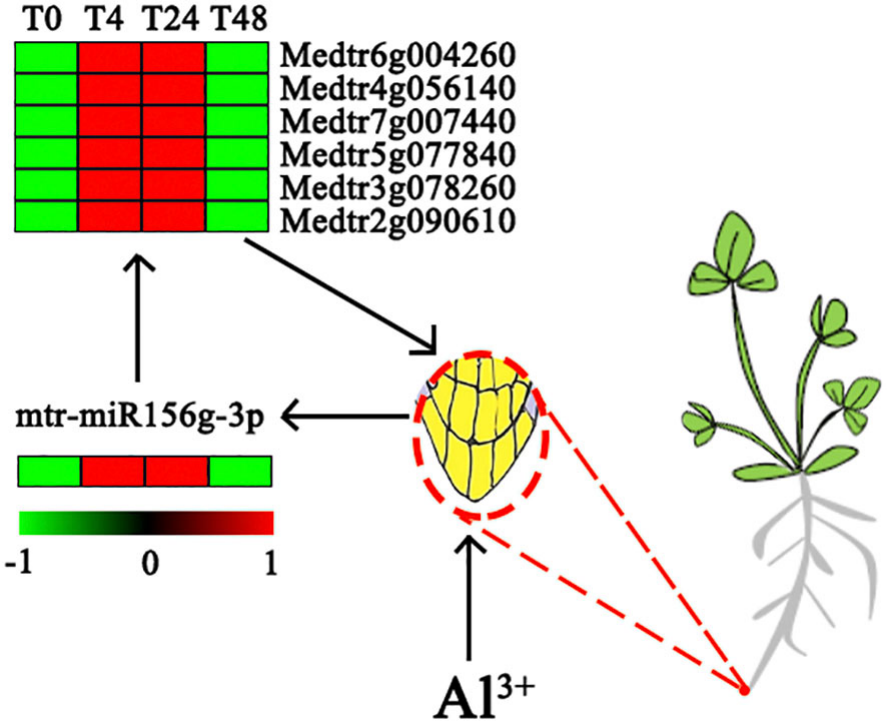
Analysis of expression patterns of miRNA and target genes affecting the root system of *M. truncatula* under aluminum stress.

miR156 is one of the most abundantly expressed and highly evolutionarily conserved miRNAs in plants (Niu et al., 2015), and the physiological processes involved in the regulation of this family of miRNAs under abiotic stress have been demonstrated in *A. thaliana* (Guan et al., 2014; Niu et al., 2015), *Panax notoginseng* (Zheng et al., 2017), *Phalaenopsis* (Zhao et al., 2019), and other plants. miR156 is highly accumulated mainly in the seedling stage of plants, and its expression level decreases during the plant growth period, which is important for plant seedling development and for improving crop productivity and stress resistance (Wang et al., 2009; Wu et al., 2009; Jerome Jeyakumar et al., 2020). In the present study, mtr-miR156g-3p was the only DE miRNA involved in the response at 4, 24, and 48 h of Al stress, and it was upregulated compared to the control. Further annotation analysis of the target genes of mtr-miR156g-3p showed that six target genes, Medtr6g004260, Medtr4g056140, Medtr7g007440, Medtr5g077840, Medtr3g078260, and Medtr2g090610, regulate the formation of the root tip cell membrane. In addition, the expression levels of the above six target genes were significantly upregulated at 4 and 24 h of Al stress (*P* < 0.05) (**Figure 5**; **Supplementary Table S10**). Therefore, these findings suggest that mtr-miR156g-3p is activated in *M. truncatula* under Al stress and further overexpresses target genes with the ability to promote cell membrane formation in root tip cells to counteract the damage caused by stress. Niu et al. (2015) found that miR156 could enhance *A. thaliana* lateral root development by regulating related binding protein genes, which is consistent with the findings of the present study. Of note, in previous studies, mtr-miR156g-3p under abiotic stress mediated the negative expression of the *SQUAMOSA PROMOTER BINDING PROTEIN-LIKE* (*SPL*) gene before regulating the expression of genes controlling root cell growth (Zheng et al., 2019; Rao et al., 2021). In contrast, there was no differential expression of genes associated with *SPL* in the present study, which may indicate that mtr-miR156g-3p in *M. truncatula* root tip tissue can directly regulate root cell growth genes. Zhao et al. (2019) found that mtr-miR156g-3p directly regulates anthocyanin formation in *Phalaenopsis* plants, and no expression of *SPL*-related genes was detected.

### 4.2 Regulation of transcription factors by miRNAs and target genes in the mechanism of Al resistance

Transcription factors play an important role in Al stress-tolerance mechanisms (Lin et al., 2021; Shu et al., 2022). The GRAS TF family is involved in a variety of biological processes such as biotic and abiotic plant stress, rootstock development, and meristem tissue formation (Wang et al., 2018). In the present study, seven target genes of four DE miRNAs were involved in the regulated expression of GRAS TFs, and all seven target genes were significantly upregulated at 4 h of Al stress, but not at 24 h and 48 h (**Table 1A**; **Supplementary Table S10**). The GRAS TF family consists of three members: GAI (GIBBERELLIC ACID INSENSITIVE), RGA (REPRESSOR OF GA1-3 MUTANT), and SCR (SCARECROW) (Fan et al., 2021), which are mainly expressed in roots and vascular cells with SHR (SHORT ROOT) and can enhance plant stress resistance (Cui et al., 2014). The MYB TF family members play an important role in plant cell wall formation, growth, and development, and are one of the major components of stress response mechanisms in plants (Sun et al., 2014). In this study, 14 target genes of 10 DE miRNAs regulated the expression of MYB TFs at 4 h of Al stress treatment, and all the 14 target genes were significantly upregulated. In contrast, no DE miRNAs regulating MYB TFs or their corresponding target genes were activated at 24 h of Al stress treatment. At 48 h, a total of two target genes of one DE miRNA were involved in the regulation of MYB TF expression, both of which were significantly downregulated (**Table 1A**; **Supplementary Table S10**). The MYB TFs are one of the largest protein families in plants (Ambawat et al., 2013), and most MYB proteins belong to the R2R3-MYB subfamily, which positively regulates salt tolerance in plants by mediating the expression of abscisic acid (ABA) and regulating cuticle formation (Wang et al., 2021). WRKY is a plant-specific zinc finger TF that is involved in various physiological processes in plants (Wu et al., 2014; Wang et al., 2022). In this study, a total of 13 target genes of 7 DE miRNAs regulated the expression of WRKY TFs at 4 h of Al treatment, and all 13 target genes were significantly upregulated. At 24 h, no DE miRNAs that regulated WRKY TFs or target genes were activated. At 48 h, one target gene of one DE miRNA was involved in the expression regulation of WRKY TFs, and this target gene was significantly downregulated (**Table 1A**; **Supplementary Table S10**). The WRKY TFs are not only involved in the regulation of various biological functions by themselves but also interact with other TFs to form a signaling network that regulates different biological processes and are an important component of the plant stress tolerance system (Wani et al., 2021). The bHLH class of TFs is the second largest class of TFs in plants and is involved in biological processes, such as light signaling, hormone and other signal transduction, root hair development, and stress tolerance mechanisms (Nakata et al., 2013). In the present study, a total of 10 target genes of 4 DE miRNAs were involved in the regulated expression of bHLH TFs, and all 10 target genes were significantly upregulated at 4 h of Al stress, whereas they were not expressed at 24 h and 48 h of Al stress (**Table 1B**; **Supplementary Table S10**). In the present study, the expression pattern of bHLH TFs was consistent with that of GRAS TFs, and both TFs play an important role in the growth and development of plant roots under stress (Nakata et al., 2013; Wang et al., 2018). Therefore, the present study suggests that related miRNAs promote the development of plant roots by regulating the expression of bHLH TFs and GRAS TFs at the early stage of Al stress in *M. truncatula*, thus enhancing the Al resistance of *M. truncatula*.

**Table 1A:** Information About Special MiRNA.

TFs family	miRNA	T0_TPM	T4_TPM	T24_TPM	T48_TPM	Regulated	Target genes
GRAS	mtr-miR156h-3p	24.35	61.27	–		up	Medtr7g027190
	novel_miR_135	119.16	202.35	–	–	up	Medtr4g133660
							Medtr4g077760
	novel_miR_182	–	202.12	–	–	up	Medtr4g077760
							Medtr4g133660
	novel_miR_36	–	202.35	–	–	up	Medtr4g133660
							Medtr4g077760
MYB	mtr-miR156h-3p	24.35	61.27	–	–	up	Medtr5g038910
							Medtr3g065440
	mtr-miR5559-5p	56.00	87.61	–	–	up	Medtr1g083630
	novel_miR_135	–	202.35	–	–	up	Medtr0193s0090
	novel_miR_143	26.28	49.27	–	–	up	Medtr2g096380
	novel_miR_180	74.14	113.42	–	–	up	Medtr6g055910
	novel_miR_182	–	202.12	–	–	up	Medtr0193s0090
	novel_miR_187	27.84	65.83	–	–	up	Medtr8g042410
	novel_miR_36	–	202.35	–	–	up	Medtr0193s0090
	novel_miR_45	70.44	112.34	–	–	up	Medtr2g100930
							Medtr3g077650
							Medtr4g057635
							Medtr3g074520
	novel_miR_9	78.60	123.77	–	–	up	Medtr7g011170
	mtr-miR397-5p	73.44	–	–	33.94	down	Medtr7g461410
							Medtr7g061330

-, indicates that the MiRNA is not expressed in the corresponding aluminum treatment group.

**Table 1B:** Information About Special MiRNA.

TFs family	miRNA	T0_TPM	T4_TPM	T24_TPM	T48_TPM	Regulated	Target genes
WRKY	mtr-miR156h-3p	24.35	61.27	–	–	up	Medtr3g085710
	novel_miR_135	–	202.35	–	–	up	Medtr8g067650
							Medtr6g053200
							Medtr1g099600
	novel_miR_180	74.14	113.42	–	–	up	Medtr6g015675
	novel_miR_182	–	202.12	–	–	up	Medtr6g053200
							Medtr8g067650
							Medtr1g099600
	novel_miR_36	–	202.35	–	–	up	Medtr1g099600
							Medtr8g067650
							Medtr6g053200
	novel_miR_45	70.44	112.34	–	–	up	Medtr7g062220
	novel_miR_9	78.60	123.77	–	–	up	Medtr3g056100
	novel_miR_193	63.10	–	–	23.93	down	Medtr4g132430
bHLH	mtr-miR172b	17.01	7.41	–	–	up	Medtr3g116770
							Medtr2g038040
	mtr-miR172c-3p	17.01	7.80	–	–	up	Medtr3g116770
							Medtr2g038040
	mtr-miR172d-3p	29.16	13.71	–	–	up	Medtr2g038040
							Medtr4g079760
	novel_miR_135	–	202.35	–	–	up	Medtr5g005110
							Medtr4g079760
							Medtr2g039620
	novel_miR_182	–	202.12	–	–	up	Medtr5g005110
							Medtr4g079760
							Medtr2g039620
	novel_miR_36	–	202.35	–	–	up	Medtr2g039620
							Medtr5g005110
							Medtr4g079760
F-box	miR2119	14.90	–	34.263	–	up	Medtr4g134390
							Medtr8g467670
							Medtr2g020070
							Medtr3g024110
	miR5213	104.43	–	170.14	–	up	Medtr5g069120
							Medtr7g079370

-, indicates that the MiRNA is not expressed in the corresponding aluminum treatment group.

MicroRNAs and target genes mediate TFs, such as GRAS, MYB, WRKY, and bHLH, which play an important role in the Al response mechanism of *M. truncatula*. However, all target genes regulating the above TFs were significantly upregulated at 4 h during the initial stage of Al stress. In contrast, no genes regulating TFs were activated at 24 h. Although some target genes of individual TFs were activated at 48 h, they were all downregulated. This study concluded that the above results could be attributed to the severe damage to the root tip tissue of *M. truncatula* with the increase in Al stress time, resulting in a large amount of cell inactivation. This reduces the ability of root tip cells to express Al tolerance genes and secrete secondary substances involved in the Al stress response. The above expression pattern of TFs was also consistent with the trend of the root physiological indicators in **Figure 1**. Notably, three miRNA target genes, novel_miR_135, novel_miR_182, and novel_miR_36, were involved in regulating the expression of the above four TFs, and they were significantly upregulated at the early stage of stress. Therefore, this study suggests that three miRNAs, novel_miR_135, novel_miR_182, and novel_miR_36, and their target genes may have potential roles in regulating Al responsive TFs.

### 4.3 Role of legume-specific miRNAs in the Al response mechanism of *M. truncatula*

miR2111, miR2119, and miR5213 are considered legume-specific miRNAs that regulate the expression of relevant defense genes when legumes are attacked by pathogens, thereby enhancing their own defense mechanisms (Kohli et al., 2014). This has been verified in plants such as *Prunella vulgaris* (Rosa et al., 2019) and *Cicer arietinum* (Kohli et al., 2014). In the present study, three target genes of miR2119 and one target gene of miR5213 were significantly upregulated at 24 h of Al stress, while their expression levels at other time points of Al stress were consistent with the treatment group **Table 1A**; **Supplementary Table S10**). Functional analysis of the four target genes revealed that they were involved in the regulation of an F-box protein containing a TIR structural domain (**Supplementary Table S8**). F-box proteins are receptors for plant growth hormones and are involved in plant defense responses (Dharmasiri et al., 2005; Navarro et al., 2006). Therefore, our findings suggest that legume-specific miR2119 and miR5213 may enhance Al tolerance in *M. truncatula* by regulating F-box protein overexpression *via* their target genes.

## 5 Conclusions

In this study, a total of 195.80 M clean reads were obtained from 12 sRNA libraries, and 876 miRNAs were identified, along with their corresponding 14,455 target genes. A total of 55 miRNAs were differentially expressed during Al stress, with different miRNAs enhancing Al tolerance in *M. truncatula* through mechanisms such as the promotion of root tip cell formation, expression of Al-resistant TFs, and regulation of defense gene expression. These findings could provide useful genetic resources for subsequent biotechnological studies, such as miRNA silencing or gene overexpression, and may inform genetic improvement programs for the development of Al stress-tolerant genotypes in legume crops.

## Data availability statement

The datasets presented in this study can be found in online repositories. The names of the repository/repositories and accession number(s) can be found at: https://www.ncbi.nlm.nih.gov/, PRJNA908067.

## Author contributions

RD and CC conceived the experiment. ZL, ZY, ZT, and QG carried it out. ZL and RD analyzed the data. RD and ZL wrote the paper. All authors contributed to the article and approved the submitted version.
